# Autologous activated platelet-rich plasma injection into adult human ovary tissue: molecular mechanism, analysis, and discussion of reproductive response

**DOI:** 10.1042/BSR20190805

**Published:** 2019-06-04

**Authors:** E. Scott Sills, Samuel H. Wood

**Affiliations:** 1 Gen 5 Fertility Center, Office for Reproductive Research, Center for Advanced Genetics; San Diego, CA, U.S.A.; 2 Applied Biotechnology Research Group, University of Westminster; London W1B 2HW, U.K.

**Keywords:** aging, fertility, menopause, ovary, reproduction

## Abstract

In clinical infertility practice, one intractable problem is low (or absent) ovarian reserve which in turn reflects the natural oocyte depletion associated with advancing maternal age. The number of available eggs has been generally thought to be finite and strictly limited, an entrenched and largely unchallenged tenet dating back more than 50 years. In the past decade, it has been suggested that renewable ovarian germline stem cells (GSCs) exist in adults, and that such cells may be utilized as an oocyte source for women seeking to extend fertility. Currently, the issue of whether mammalian females possess such a population of renewable GSCs remains unsettled. The topic is complex and even agreement on a definitive approach to verify the process of ‘ovarian rescue’ or ‘re-potentiation’ has been elusive. Similarities have been noted between wound healing and ovarian tissue repair following capsule rupture at ovulation. In addition, molecular signaling events which might be necessary to reverse the effects of reproductive ageing seem congruent with changes occurring in tissue injury responses elsewhere. Recently, clinical experience with such a technique based on autologous activated platelet-rich plasma (PRP) treatment of the adult human ovary has been reported. This review summarizes the present state of understanding of the interaction of platelet-derived growth factors with adult ovarian tissue, and the outcome of human reproductive potential following PRP treatment.

## Background

An important aspect of successful IVF is the surgical recovery of an adequate number of oocytes for prompt and monitored fertilization (usually via ICSI). It is the paucity of this essential egg contribution which is typically foreshadowed by laboratory tests indicating diminished reserve [1]. The availability of mature oocytes for IVF is in fact the closing chapter in a long signaling narrative within the ovary, evolving from multiple events. This seems to begin with the development of the ovary itself, reaching a conclusion at the crescendo moment of ovulation. Throughout this journey, the oocyte precursor which acts as a central passenger is accompanied by supporting somatic cells to enable survival and eventual maturation of the egg. For example, the growing germ cells gradually become vested with epithelial elements known as ‘ovigerous cords’ comprising pregranulosa cells. As ovarian development concludes, these ovigerous cords splinter off into individual primordial follicles—consisting of the oocyte surrounded by its companion single layer of granulosa cells.

## The ovary: its development and actions

Acting primarily to sustain oocyte development and to produce maturational hormones necessary for puberty, the adult ovary is the key regulator of the reproductive cycle and pregnancy over the course of the female reproductive career. These myriad functions require a constant cascade of remodeling and regression, entailing considerable biochemical and tissue reorganization [2]. Of note, several pathological ovarian conditions including polycystic ovary syndrome, premature ovarian insufficiency/failure, and ovarian malignancies have all been linked with disturbances in how cells within the ovary behave. Early work with several novel interventions has suggested ways to improve ovarian response during IVF, but, with uncertainty. In fact, more questions have emerged as findings from pilot studies are assessed. In the meantime, any effort to enhance or extend fertility must be predicated on the fullest possible knowledge of the cell and tissue remodeling processes which occur in the ovary.

An understanding of the developmental origins of the ovary may be guided by observations from comparative anatomy with related structures, particularly testis and adrenal. Yet in the special case of the ovary, there is one characteristic which sets it apart from most other physiologic systems—unlike other female endocrine organs, the ovary undergoes special functional and morphological modifications at puberty. Even during early embryological development, ovarian morphogenesis is far from simple. As mesonephric derivatives, the gonads follow distinct developmental pathways for males and females but do transit a brief phase of bipotentiality before committing to their developmental destiny. Moreover, some ovarian components actually originate outside the organ and arrive later as imports [3]. This would include primordial germ cells (PGCs) (from yolk sac) and certain immune cells (from dorsal aorta); sources for some somatic cell types still remain unclear [4].

This interplay between germ cells and somatic cells appears critical, as fragmentation of ovigerous cords into independent follicles does not occur in the absence of germ cells. Thus proper female differentiation of ovarian somatic cells is modulated by the oocyte itself, as the latter appears to inhibit the testis-differentiating sequence to conserve the fate of adjacent pregranulosa cells (instead of developing into Sertoli cells, for example). In this milieu, primordial follicles continue to be recruited into the growing follicle population to develop through primary, preantral, antral, and preovulatory stages before being released at ovulation. The oocyte also governs the functional differentiation of granulosa cells, preventing their premature maturation into luteal cells in the final stages of growth [5].

Parallel to the tandem, symbiotic relationship between germ cells and somatic cells coordinating their actions to yield follicles, evidence now exists to show that some germ cells are present on or near the ovarian surface [6,7]. With lateral stromal expansion under the ovarian capsule, the once ‘open’ ovigerous cords eventually close and become isolated from the surface, thus marooning some epithelial cells and some egg precursors at the ovarian surface [8]. What is the developmental purpose of this process, and what evolutionary advantage is conferred by it? While the function of these stranded germ cells is not known, some seem to be lost from the ovarian surface into the periovarian space [9,10] or undergo local atresia. It could be that these are the source of GSCs (germline stem cells) which have been isolated from surface or outer cortex regions of the ovary [11].

## Ovarian GSCs: sources and destinations

The reason these cells matter to current fertility practice is that, at least for the past half century, clinicians have worked under the assumption that the entire reserve of oocytes is fixed at birth. Human ovaries are not supposed to have the potential to receive any deposits to this account after this time. Moreover from this oocyte endowment, only withdrawals are possible over a lifetime until the balance is depleted, reaching zero at menopause [12]. This classical ovarian reserve concept met a serious challenge in 2004, and reignited a debate regarding whether oogenesis might be possible in mammals far into adulthood [13]. Specifically, putative GSCs (oogonial stem cells) have been reported to exist in the ovaries of adult humans [11], mice [14], and rats [15]. The first description of renewal of germ cells in postnatal mice ovaries was more than 10 years ago, after examining changes in follicle numbers with age [13]. These investigators subsequently found expression of germline markers in bone marrow-derived cells [16]. Interestingly, bone marrow and peripheral blood transplantations resulted in recovery of oocyte production in wild-type mice sterilized by chemotherapy and ataxia telangiectasia-mutated mice. From this, it was concluded that bone marrow and peripheral blood might be a potential source of female germ cells that could permit egg production in adulthood.

Curiously, a parabiosis experiment [17] failed to support this finding, in which the vasculature of wild-type mice was surgically grafted to that of transgenic mice expressing green fluorescent protein (GFP) under the control of the β-actin promoter. Even though high levels of blood cell chimerism were noted, no GFP-positive germ cells were ovulated in the non-transgenic mice [17]. Further research focused on effects of bone marrow transplantation from TgOG2 transgenic mice with germline-specific expression of GFP (Oct4-GFP) into recipients depleted of ovarian follicles due to cyclophosphamide and busulfan exposure [18]. Bone marrow-derived germ cells have been found in primordial and immature growing follicles, but these did not advance to the ovulatory stage. It was also shown that bone marrow-derived germ cells were not CD45^+^ monocytes, and Oct4-GFP is not exclusively specific to germ cells, as this marker is also expressed in other adult stem cell populations and tumors [17,19]. Thus, the possibility exists that GFP+ cells noted in recipient mice were actually macrophages [18] since they lacked the typical morphology of oocytes and Oct4+ macrophages seen previously in rabbit tissue in association with atherosclerotic plaque [20]. Such apparently conflicting findings could be explained by considering that transplanted bone marrow-derived (or blood-borne leukocytes) do not actually replace ovarian germ cells, but rather support their development and recovery from radio- or chemotherapy [19].

It is now recognized that the immune system plays an important supportive role in ovarian function, particularly with respect to follicle development [21,22]. Recent work has found a population of CD4^+^CD25^+^FOXP3^+^ Treg cells to be especially relevant, and these cells from females exhibit a more potent suppressive function than similar cells obtained from males [23]. Interestingly, this sex-specific effect can be reversed if males are grafted with ovarian tissue [24], demonstrating an antigen-specific Treg suppression and the need for sustained presence of the cognate tissue antigen to produce ovary antigen-specific Treg cells. Dysfunction of normal immunomodulatory function in the ovary—particularly loss of Treg cells—has been suggested as a cause for premature ovarian insufficiency in some cases [25].

So where do eggs ultimately come from? It seems unlikely that oocytes arise from a hematopoietic stem cell source. This mechanism was refuted by data from a study using a ‘molecular clock’ method to estimate the number of cellular mitotic divisions since arising from zygote stage [26]. The approach followed specific (somatic) mutations to develop lineage trees, predicated on the concept that spontaneous mutations in DNA can be used to count the number of mitotic divisions (‘depth’) a cell has undergone since soon after fertilization; in this way mutation patterns in multiple loci can reveal the lineage relations among individual cells. Thus an assembly-line model of oocyte activation was advanced, whereby the earliest oocytes eligible for ovulation are also those which enter meiosis first [27]. Importantly, the ‘mitotic age’ or ‘depth’ of oocytes was found to be different across mesenchymal and hematopoietic stem cells of bone marrow origin [26], making it difficult to show that oocytes are seeded from bone marrow cells.

Almost 10 years ago, a key development was reported when a population of mitotically active cells discovered in immature and adult mouse ovaries were successfully manipulated *in vitro* to evoke germline characteristics [14]. The isolation of such cells, however, was insufficient to prove definitively that they are involved in postnatal oogenesis. Indeed, the ovary-derived cells were different from bone marrow-derived cells, exhibiting stable expression of germline markers such as Oct4, MVH, Dazl, Blimp1, Fragilis, Stella, and Rex1 [14]. Using a transplantation model to repopulate cells in murine ovaries affected by chemotherapy exposure, it was shown that these cells might be ovarian GSCs. Moreover, *de novo* oocytes were identified and were capable of fertilization, resulting in birth of live offspring carrying a traceable genetic marker introduced into the cells before transplantation. Mating of this generation with wild-type mice produced transgenic offspring, which inherited the marker via transgene germline transmission [14]. To explain this, it has been theorized that the ovary capsule (epithelium) might be the source of GSCs, because immunohistochemical studies have found cells double-positive for both mouse vasa homolog (MVH) and the proliferation marker 5-bromodeoxyuridine [13,14]. Using a female transgenic mouse model expressing GFP regulated by Oct4 promoter, a GFP-positive cell population was found near ovary epithelium in adult mice [28]. Such GFP-positive cells were stable in culture for up to 1 year and expressed several germ cell-specific markers (GCNA [germ cell nuclear antigen], cKIT, MVH) with sustained telomerase activity. The culture of these GSCs with granulosa cells of neonatal mice did enable development of follicle-like structures, but their functionality remains untested.

Cultured GSCs expressing GFP from neonatal and adult mice have been transferred into chemotherapy-pretreated recipient mice, producing transgenic F1 and F2 offspring [29]. Transfection of GSCs with recombinant viruses carrying Oocyte-G1, Dnaic2 (mouse dynein axonemal intermediate chain 2) or liposome-mediated transfection with an Oocyte-G1 knockdown vector, yielded heterozygous offspring after transplantation into chemotherapy-pretreated mice. No transgenic offspring were observed after transplantation of short-term cultured and GFP-transfected oocytes, providing evidence that the transgenic offspring following transplantation of GFP-positive GSCs were not from oocytes [29,30]. Comparisons of gene expression profiles among embryonic stem cells, PGCs, GSCs (fresh isolates), and cultured GSCs from adult mice revealed that the profile of PGCs was highly concordant with embryonic stem cells, whereas fresh GSCls did not express the pluripotency-associated genes *Zfp296* (encoding zinc finger protein 296), *Utf1* (undifferentiated embryonic cell transcript factor-1), *Nanog*, and *Sox2* (SRY box 2) [31]. Cultured GSCs did resemble PGC markers however, as Zfp296, Utf1, Nanog, and Sox2 all were present. Interestingly, these cultured GSCs also weakly expressed Stra8 (stimulated by retinoic acid 8), a marker of cell entry into meiosis.

The efficiency of conversion for such cells into oocytes appears very low [32]. While it may be that less than 1% of seeded GSCs spontaneously differentiate into oocyte-like cells expressing Stra8, this oocyte conversion yield was doubled with the addition of BMP4 (bone morphogenetic protein 4), known to assist induction of PGCs in mouse embryos [33]. These oocyte-like cells demonstrated much higher Stra8, Msx1 (muscle segment homeobox 1) and Msx2 expression [32], regarded as BMP-responsive genes in human and mouse fetal ovaries [34,35].

Given the importance of these findings it was not entirely surprising that other researchers critiqued the nature and consistency of cells isolated, as well as the laboratory protocol used to produce them [36]. An improved fluorescence methodology was later suggested to isolate and purify GSCs [11], in support of earlier work which concluded primitive germ cells could produce fertilizable oocytes and embryos. GSCs have also been derived from adult human ovaries, cultured *in vitro*, and shown after injection into human ovarian cortex cells to develop into what look like immature oocytes (validated by gene marker labeling); these were later enclosed by granulosa cells to form follicles [11].

It should be noted that there is no agreement on the preferred technique to isolate GSCs. Relying on DDX4/MVH expression to isolate and purify GSCs is problematic since DDX4 (a type of RNA helicase) can also be present in germ cell cytoplasm [37]. Cells isolated without permeabilization have expressed other germline markers like Dppa3, Prdm1, Dazl, Tert (telomerase reverse transcriptase), and Ifitm3 (Fragilis), but not oocyte-specific markers such as Zp3 (zona pellucida sperm binding protein 3), Nobox (newborn ovary homeobox protein), or Gdf9 (growth differentiation factor 9). This suggests the existence of ‘immature’ germline cells in the ovary, capable of expressing DDX4 or domains of DDX4 on the cell surface. DDX4 might become silenced in undifferentiated GSCs by insertion into the cell membrane, and after commitment to the oocyte fate, DDX4 is no longer externally expressed [31]. Other methods to identify and isolate (murine) GSCs with improved efficiency using antibodies and antibody-assisted magnetic-bead sorting have also been reported [38]. In any case, female GSCs obtained from prepubertal or neonatal mice have been induced to become pluripotent embryonic stem-like cells under specified culture conditions [39] and these GSCs have characteristics similar to male GSCs/spermatogonial stem cells [40].

## Pathways to the oocyte

The developmental lineage of human eggs has received considerable investigative attention [41]. Their cellular ancestor (the PGC) is known to appear very early in embryonic life. In mice, precursors of PGCs have been identified as early as embryonic day (E) 6 or 6.5 [42]. Such PGCs precursors develop under the control of signals including BMP2, 4, and 8, and are characterized by expression of PR domain containing 1 (PRDM1 or BLIMP1), PRDM14, and up-regulation of Fragilis (also known as IFITM3 or interferon-induced transmembrane protein 3). Within the first week of embryo development (E7), small clusters of PGCs stabilized by E-cadherin arrive posterior to the primitive streak in the extraembryonic mesoderm. PGCs express TNAP (a non-specific alkaline phosphatase) and DPPA3 (developmental pluripotency associated 3, also known as Stella) at about this time. Soon afterward PGCs migrate to the hindgut and, via dorsal mesentery, into the developing genital ridges. During this migration process, PGCs still express TNAP but also OCT3/4 (octamer-binding transcription factor 3/4; also known as POU5F1), the proto-oncogene cKIT, and SSEA (stage-specific embryonic antigen) 1 and 3. By the time the murine embryo enters its 12th day (E12), most PGCs have already arrived at the genital ridges. Human PGCs are first identified at gestational week 3 (E21) in the dorsal wall of the yolk sac, near the developing allantois [43]. By the time genital ridges develop by week 5, the PGCs have migrated from the hindgut to the dorsal mesentery and then further laterally, to colonize these structures.

There is now general agreement that *in vitro* differentiation of PGCs into gametes is indeed the crucial step and remains a major bottleneck [17]. PGCs are first seen in the proximal epiblast around E7 in mice, migrate via the aorta-gonad-mesonephros region, ultimately settling in the gonadal ridge to proliferate in large number (to approximately 25000 cells) as the second week approaches. There exists an intriguing overlap between PGCs migration along the dorsal mesentery and primitive hematopoiesis which is initiated at about the same time [18]. Being pluripotent, PGCs can produce both germ cells as well as hematopoietic cells. Prior to the second week, genital ridge PGCs stop dividing (female cells enter meiosis; male cells show mitotic arrest). Oogonia are formed in females next, whereas in males these become gonocytes.

At birth, gonocytes undergo rapid proliferation to form spermatogonia which further proliferate and differentiate into spermatocytes, next undergoing meiosis and later forming sperm. Crucially, a small number of spermatogonial stem cells with the ability to self-renew and further differentiate into sperm remain in the testis throughout adult life. A related cell set with small diameter (3–6 μm) is notable for long telomeres and pluripotent markers such as Oct-4, Nanog, Rex-1, SSEA-1 (in mice) and SSEA-4 (in humans); these are termed very small embryonic-like stem cells (VSELs). These pluripotent VSELs have been reported in adult tissues including gonads; they are relatively quiescent, have sufficient resilience to survive radiation and remain present in senescent, non-functional gonads. VSELs can be sorted as Sca^+^ LIN-CD45^−^ in mice and as CD133^+^LIN-CD45^−^ in humans. As with embryonic stem cells, VSELs stain positive for alkaline phosphatase, have a distinct spherical shape with a large nucleus surrounded by a thin rim of cytoplasm and high nuclear:cytoplasmic ratio. Interestingly, mouse bone marrow VSELs have been shown to have transcriptionally active chromatin elements for both Oct-4 and Nanog promoters [44]. Their pluripotent state is shown by their ability to self-renew and differentiate *in vitro* into all three germ layers in both mice and humans [45,46]. VSELs mobilize in circulation in response to injury to regenerate damaged tissues and also in response to G-CSF treatment [47–49].

Being functionally and developmentally equivalent to PGCs (as natural precursors to gametes), VSELs may spontaneously differentiate into gametes *in vitro*. Niche cells such as Sertoli cells in the male (and mesenchymal cells in the female) can be transplanted and restore gonadal function by providing paracrine support to endogenous VSELs. Such an approach has been used successfully in animal studies and has resulted in a livebirth in a woman with premature ovarian failure [50]. These VSELs are the PGCs which migrate to the gonadal ridge during early embryonic development and persist long after the postnatal period [43].

Such similarities notwithstanding, there are some key contrasts between migrating PGCs (15–20 μm) and VSELs (3–6 μm); additional research is needed to establish whether VSELs are more developmentally primitive than PGCs. It is plausible that PGCs could actually be a precursor to pluripotent stem cells *in vitro*, although they do not seem to behave as stem cells *in vivo*. Indeed, later in fetal development the true stem cell population of SSCs appears in the testis and divides throughout life, yielding ongoing spermatogenesis. The ovary could have comparable cells with stem-like characteristics capable of differentiating into oocytes, yet controversy remains on this point [51,52].

Expression of pluripotency transcription factors is lost after gastrulation in most epiblast stem cells, and these develop further into somatic structures. VSELs present in adult tissues might actually be PGCs, or their precursors [53]. Such a hypothesis is supported from observations that both PGCs and VSELs are pluripotent and relatively quiescent, and this shared quiescence is secondary to similar epigenetic modification of (paternally) imprinted genes including *Igf2-H19* and *KCNK1p57*. In addition, both PGCs and VSELs express Stella, Fragilis, Blimp1, MVH; late migrating markers specific to PGCs such as MVH, Dppa 2, Dppa4, Sall4 are also expressed by VSELs. VSELs also express several miRNAs that dampen Igf-1/Igf-2 signaling in these cells (mir681, mir470, mir669b) as well as up-regulate expression of p57 (mir25.1, mir19b, mir92). Similarities continue in that VSELs have also been found to express functional receptors for genes involved in PGC development into gametes. VSELs found in gonads and bone marrow may explain the observed plasticity and the ability of bone marrow cells to differentiate into germ cells [54]. Given the developmental origin of VSELs, their proliferation, like PGCs, is controlled by DNA methylation status of several imprinted genes (*e.g*., Rasgrf1, H19, and Igf2). During cellular senescence such proliferation-repressive epigenetic factors gradually disappear. This causes an increased sensitivity to Ins/Igf signaling, which in turn leads to depletion of VSELs [55].

Interestingly, a direct developmental link between PGCs and hematopoiesis may also exist [45]. Considerable overlap exists among chromosomal aberrations seen in germline tumors and leukemias/lymphomas, suggesting they share a common clonal origin with precursor VSELs. Thus it could be that a VSEL population exists in adults, undergoing hematopoiesis in bone marrow, and gametogenesis in the gonads. This concept fundamentally challenges accepted thinking that PGCs migrate exclusively to the gonadal ridge to yield germ cells. Rather, migration to various tissues by PGCs may occur, persisting well into adulthood to serve as a reserve pool for tissue committed stem cells [54]. Yet defining, locating, and isolating the cells that fulfil pluripotency criteria remains controversial, underscoring the importance of characterizing the cellular phenotype of these cells more completely [56]. But even when reliable techniques to harvest such cells become available, what will be the next step?

## Follicular development, recruitment, and ovulation

**Figure 1:**
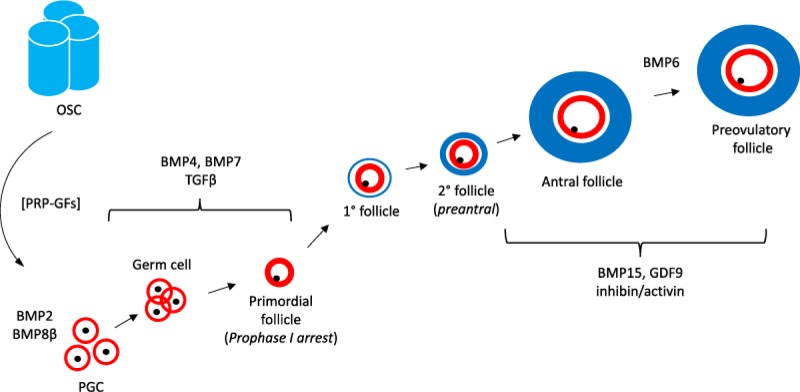
Recruitment and growth of oocytes, from PGC stage through mature follicle, illustrating various growth factors mediating development Known components of this sequence include granulosa precursors (red), theca compartment (blue), and germ cells (black). Upstream contributions by ovarian stem cells (OSC) may be possible under conditions enabled by growth factors released by platelet-rich plasma (PRP-GFs). Other relevant regulators are BMP2, BMP6, and BMP8β, which are involved in cytokine–cytokine receptor interactions; and Transforming growth factor β (TGF-β), which activates various substrates and regulatory proteins inducing transcription of genes for differentiation, chemotaxis, and proliferation. Later direction is under control of BMP15, a paracrine signal exclusively expressed in ovarian tissue which is involved in oocyte and follicular growth, as well as GDF9, a down-regulator of inhibin-A and promoter of further follicular maturation.

Follicles within the ovary may be tracked back (anatomically) to the differentiation of the oogonia within primordial follicles (see Figure 1), although steps essential to activation of primordial follicles (physiologically) may revert even further and are incompletely characterized. What is known is that some follicles join a gradually enlarging primary follicle cohort, thereby beginning a journey which terminates either at ovulation or follicular atresia. Along this developmental course, what switching mechanisms determine the destiny of each member? It appears that this sequence is regulated by intrinsic factors generated by somatic elements of the ovary, especially granulosa and theca cells. Operating in concert, these two compartments produce signals required for the follicle to advance to late preantral or early antral development. The endocrine effects of FSH and LH are needed to sustain further follicular growth; atresia is generally the consequence of the failure to receive or process such gonadotropin signaling. As a follicle approaches its periovulatory phase, other players join the signaling orchestra including prostaglandins, steroids, and proteins of the epidermal growth factor family. While the precise measure and contribution of each signal member require additional study, it is clear that the achievement of follicle maturation entails a sophisticated program of regulatory mediators of both somatic and germ cell origin [57]. Ovarian surface epithelium derives from the mesodermal lining of the intraembryonic coelom and nearby areas where the gonad is formed [58,59]. However, when the bovine fetal ovary first develops it is not at first vested with a defined surface epithelium underlaid by a basal lamina (as observed in the adult), except at the base of the ovary where it arises from the mesonephros [60,61].

It may be that the nascent ovary begins as a cluster of gonadal ridge epithelial like (GREL) cells, which themselves proliferate from the mesonephric surface epithelium, in a process that is also associated with degradation of the basal lamina. This allows the PGCs then to associate with adjacent GREL cells. It has been observed that the mesonephric surface epithelium is single-layered, except where gonadal thickening occurs [62]. The stroma does not penetrate into the ovary until later, and GREL cells on the surface eventually become epithelium only after the stroma has expanded to just underneath the GREL cells [60]. Even though a defined surface epithelium is lacking in the early ovary, the ovarian hilum is an exception where a mesonephric protrusion exists, covered by surface epithelium with a subepithelial basal lamina and epithelial–stromal interface which directly originates from the mesonephros [60]. The rest of the ovary derives its surface epithelial cells from GREL cells. This may be relevant since the surface epithelium of the adult mouse ovary is not uniform, as the base (hilum) of the mouse ovary has stem cells with greater oncogenic potential compared with cells at other ovarian surface locations [61,62]. It is plausible that these differing developmental origins of ovarian epithelial cells (hilum vs. elsewhere) contribute to the varied behavior of epithelial cells, depending on their address within the ovary.

At each ovulation the ovarian epithelium experiences injury by rupture, with the continuous layer of surface epithelium (and underlying tunica albuginea) sustaining repetitive damage. It is assumed that stem cells in the remaining surface epithelium are involved in repairing this rupture; murine studies have implicated stem/progenitor cells in the ovarian surface epithelium as central to this process [63]. Specifically, pulse-chase experiments using 5-bromodeoxyuridine and transgenic mice were able to show a population of long-term label-retaining cells in the surface epithelial layer. Although dormant before ovulation, these cells activated and began replicating at the follicular margins soon afterward, indicating that these cells were contributing to repair and remodeling processes.

Mesenchymal cells in the tunica albuginea of the adult ovary can undergo a mesenchymal–epithelial transition into ovarian surface epithelium cells, which differentiate sequentially into primitive granulosa and germ cells. These structures have now been shown to assemble in deeper ovarian cortex to form new follicles, replacing older (atretic) primary follicles. Such follicular renewal has been reported in rat ovaries, and human oocytes can differentiate from ovarian surface epithelium in fetal ovaries *in vivo* and from adult ovaries *in vitro*. Thus the pool of primary follicles in adult human ovaries does not represent a static, but rather a dynamic, population of differentiating and regressing structures [6].

What signaling events might call progenitor cells (or stem cells) forward within the ovary to activate a post-ovulatory local tissue injury repair? And which markers seem most relevant to study in this regard? It has been suggested that WNT/β-catenin are involved in this process by differentiation of progenitor cells in the ovarian surface epithelium [64]. Specifically, transgenic mice with a β-catenin/TCF (T-cell factor)-responsive lacZ reporter gene were studied to help identify WNT-activated cells. Interestingly, lacZ expression occurred in the undifferentiated gonad, but after sex determination, expression was limited to the female gonad—a pattern agreeing with the membranous localization of β-catenin in embryonic murine gonads [65]. Furthermore, this ovarian surface epithelium cell gene expression declined after birth to a population of just 0.2% of the total surface epithelial cell population. This decline was not secondary to apoptosis or reduced proliferation, but rather from lacZ-positive cells differentiating into lacZ-negative cells. Thus, lacZ-positive cells (active β-catenin/TCF signaling) in the ovarian surface epithelium seem to act as stem cells, capable of contributing to repairing ovarian surface microtrauma. In addition, WNT4 and RSPO1 up-regulate the adult stem cell marker LGR5 in developing mouse ovaries, again suggesting that this pathway directs stem cell activity at the ovary surface [66]. A population of cells has been isolated by flow cytometry from the ovarian surface epithelium of adult mice [67], expressing high levels of mRNA for the hematopoietic stem cell marker lymphocyte antigen 6 complex, locus A (LY6A). Constituting only 2% of the total surface epithelial cell population, this LY6A+ subpopulation is detectable after approximately 4 weeks. In contrast, LY6A− cells proliferated much earlier, in the first 7 days. Moreover, a process seen in stem cells known as spheroid formation was higher in LY6A+ cells compared with other surface epithelial cells. LY6A+ cells existed in the surface layer and were not in direct contact with any other ovarian structures such as follicle walls or corpora lutea. Such cells appeared more cuboidal compared with the remaining surface epithelial cells, and additionally, oocytes of primordial follicles were LY6A+. Since there is increasing evidence for the existence of GSCs on the surface of murine ovaries [38], any LY6A+ cells detected in ovarian tissue sections could be GSCs rather than progenitor cells/stem cells of ovarian surface epithelium.

Cells have been identified in the hilar region of postnatal mouse ovaries which show classical stem cell characteristics such as expression of ALDH1 (aldehyde dehydrogenase 1), LGR5, CD133 (cluster of differentiation 133), CK6B (cytokeratin 6B), and LEF1 (lymphoid enhancer-binding factor 1), as well as long-term survival/proliferation and spheroid formation in culture [61]. Importantly, the previously noted pulse-chase experiments (using 5-bromodeoxyuridine labeling) have provided evidence that these cells are specifically activated to repair the rupture/injury at the surface of the ovary following ovulation, apparently to seal off the irregular surface at the site of ovulation [62]. LGR5 expression was located on the surface and subsurface region in the fetal mouse ovary, although this was confined to the surface epithelium by postnatal d7 and in adult mice. Additional research will be needed to establish if LGR5+ cells are confined to specific epithelial areas of the ovarian hilum [61], or more widely distributed throughout the entire ovarian surface [66].

Human adult ovary research has shown that most (>75%) of surface epithelial cells express the known stem cell marker NANOG, secreted frizzled related protein 1 (SFRP1), LIM homeobox 9 (LHX9), and ALDH1A2, yet only 25% of ovarian surface epithelial cells were ALDH1A1+ [4]. Assuming these specialized cells are present in the adult human ovary, an important question which remains to be examined is this: Given what is currently known about surface markers, under what conditions might discrete signaling be produced to evoke differentiation of any precursor cell(s) to become functional *de novo* oocytes?

## The platelet signaling milieu

Relevant parallels exist between wound healing and ovarian tissue repair following capsule rupture at ovulation, and some molecular signaling events which might be necessary to reverse the effects of reproductive aging seem congruent with changes occurring in tissue injury responses elsewhere [68,69]. The interaction between platelets and plasma proteins—notably fibrin formed from fibrinogen by thrombin—causes fibrin clot formation, itself a reservoir of growth factors. These are discharged into plasma from α-granules of platelets when they are activated during wound healing and tissue regeneration. Platelet α granules [70,71] contain numerous cell signaling moieties directly involved in tissue repair such as HGF, SDF-1, adenosine diphosphate, serotonin, and sphingosine-1-phosphate; these can promote survival signals for vascular endothelial cells and SMCs (smooth muscle cells) at sites of vascular injury [72–74]. Transforming growth factor β isoform 1 (TGF-β1) is of pivotal importance given its actions on cell proliferation, angiogenesis, and extracellular matrix deposition [75]. One application of this may be seen in improved endothelial regeneration observed following injection of platelet microparticles in a mouse carotid artery injury model [76]. Platelets are also known to influence certain progenitor cell actions following tissue insult. For example, SDF-1 secreted by activated platelets support CD34-positive progenitor cell recruitment to arterial thrombi and differentiation of endothelial progenitor cells *in vivo* [77]. In the setting of myocardial infarction, platelet-derived SDF-1 was related to the number of circulating progenitor cells and was associated with restoration of left ventricular function and an improved prognosis. Formation of circulating platelet/CD34+ progenitor cell co-aggregates has been reported in patients with acute coronary syndromes, which was associated with a significantly decreased myocardial infarct size and better left ventricular function, as seen with cardiac magnetic resonance imaging at a 3-month follow-up [78–80]. However, platelet-induced differentiation of CD34-positive progenitors into mature foam cells and endothelial cells has been described in an *in vitro* co-culture system [81], which may be of particular relevance for development of atherosclerotic vascular lesions. Injection of autologous PRP (platelet-rich plasma) may terminate or even reverse the progress of early disc disease in the rabbit, which may be associated with the role of multiple growth factors of PRP in regulating cell function, improving tissue microenvironment, and/or modulating tissue regeneration [82].

**Figure 2:**
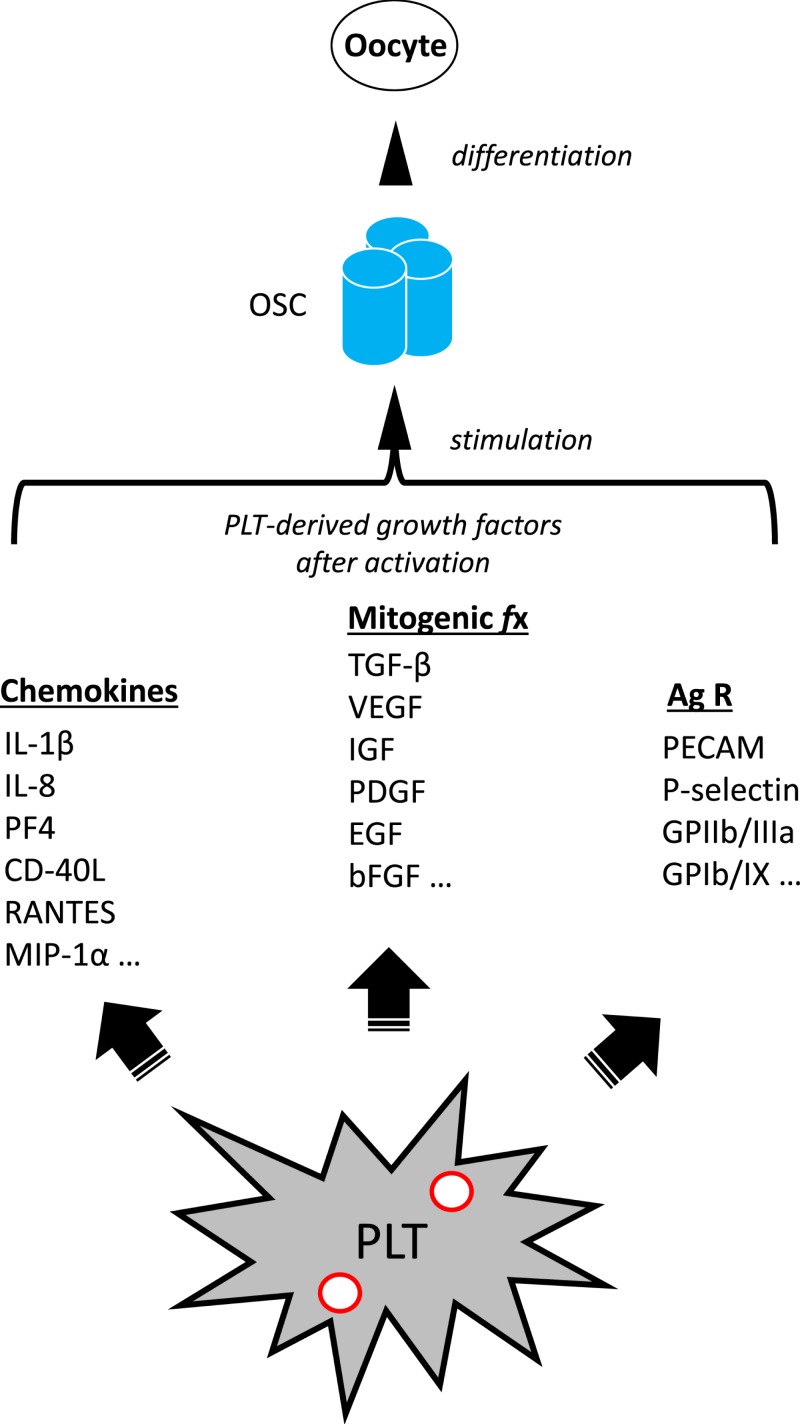
Proposed mechanism of action for alteration of adult ovarian function by application of activated PRP Autologous activated PRP sample generates an enriched platelet (PLT) substrate collected by peripheral venipuncture. PLT combination with calcium gluconate achieves activation of α granules [red circles], which subsequently initiates release of at least three classes of molecular mediators. These include chemokines such as Interleukin-1β (IL-1β), a central inflammatory mediator involved in cell proliferation, differentiation, and apoptosis; Interleukin-8 (IL-8, also known as neutrophil chemotactic factor) which coordinates migration toward sites of injury or infection and is a promoter of angiogenesis and improved tissue perfusion; Platelet factor 4 (PF4), a versatile chemotactic protein with high affinity for heparin, involved in platelet aggregation and selective antimicrobial activity; Ligand of CD40 (CD-40L), a potent inducer of inflammatory processes by enhancing interactions among platelets, leukocytes, and endothelium; a protein known as Regulated after Activation of Normal T-cell Expressed and Secreted (RANTES), itself a useful marker for PLT activation which strongly attracts monocytes; Macrophage inflammatory protein 1-α (MIP-1α), which conditionally triggers migration and signaling cascades to mediate cell survival and proliferation; Platelet-associated cellular mitogens include TGF-β, which activates different downstream substrates and regulatory proteins inducing transcription of multiple target genes for differentiation, chemotaxis, proliferation and activation of immune system cells; Vascular endothelial growth factor (VEGF), a signal protein stimulating blood vessel formation; Insulin like growth factors (IGFs) a group of proteins with close homology to insulin required for cell stimulation and communication with the local environment; Platelet derived growth factor (PDGF), critical in growth of blood vessels from extant nearby capillaries, mitogenesis and proliferation of mesenchymal cells including fibroblasts, osteoblasts, tenocytes, vascular SMCs and mesenchymal stem cells; epidermal growth factor (EGF), a central element in cellular proliferation, differentiation, and survival; Basic fibroblast growth factor (bFGF), a mediator with broad mitogenic and cell survival activities, and is involved in a variety of biological processes, including embryonic development, cell growth, morphogenesis, tissue repair, tumor growth and invasion function. Platelet expressed antigens include Platelet endothelial cell adhesion molecule (PECAM), which plays a key role in removing aged neutrophils from circulation; P-selectin which contributes to initial recruitment of leukocytes to injury sites during inflammation; Glycoprotein IIb/IIIa, part of the integrin complex found on platelets aiding in platelet activation; and Glycoprotein Ib and IX (GPIb/IX) which binds von Willebrand factor, allowing platelet adhesion and platelet plug formation at sites of vascular injury. PRP is placed inside the adult ovary (by direct ultrasound-guided needle injection) thus permitting these signaling elements access to ovarian stem cells (OSCs) as discussed by Johnson et al. (2004). PLT-derived moieties then trigger or enable differentiation of these OSCs. Subsequently, reduced serum FSH and/or higher post-treatment levels of serum AMH have been observed clinically, consistent with improved or ‘re-potentiated’ ovarian function.

Such platelet-derived growth factors are large, hydrophilic molecules with a molecular weight above 15 kDa. Unlikely to penetrate skin in sufficient quantities to be clinically significant [83], these growth factors must be injected directly into ovarian tissue to attain any meaningful therapeutic effect. Once deposited within the ovary, growth factor ligands have an opportunity to interact with receptors and regulators to influence cell differentiation outcomes (see Figure 2). An understanding of ovarian stem cell biology as outlined previously, and how this may be modulated should enhance understanding of ovary biology in general, and PRP actions in particular. For example, the growth factors produced from PRP represent a diverse group of regulatory proteins which attach to cell membrane receptors mediating important chemical messages. Via this interaction, they enable inter- and intracellular signaling pathways to govern cell growth, proliferation, and differentiation. Unlike hormones, these growth factors show quite circumscribed activity, physiologically relevant only in very close proximity to their release site. These local effects include mitogenesis, angiogenesis, chemotaxis, and formation of the extracellular matrix and even controlling release of other growth factors [83,84].

Previous research has revealed multiple critical roles of these growth factors and their receptors in embryonic and postnatal development. PDGF was originally identified in platelets and in serum as a mitogen for fibroblasts, SMCs and glia cells in culture. PDGF has since expanded to a family of dimers of at least four gene products, whose biological actions are mediated through two receptor tyrosine kinases. These products of activated platelets seem to act upon specific populations of progenitor cells that yield several different cell types with distinct functions in a variety of developmental processes. Given the wide scope of action, it is plausible that PRP elements might supply the requisite signal(s) needed to induce precursor or stem cell differentiation into a mature oocyte.

This inference finds support from earlier work which showed rescue from developmental arrest depends on PDGF (and other platelet-derived mediators like IGF-1), where these cytokines trigger DNA synthesis and cell-cycle specific proto-oncogenes *fos* and *myc* [85] with entry into mitosis within 24 h [86,87]. Studies on PDGF have led to an understanding of how cells detect a gradient of attractant and crawl toward it [88]. Guidance of cell migration during ovarian development shows mechanistic overlap with axon pathfinding, with some guidance cues used for both axon pathfinding and cell migration [89]. This role of PDGF in guiding cell migration has been investigated directly *in vivo* [90]. The migration of somatic (border) cells in Drosophila was chosen as model for directional migration in a genetically tractable system, where cells were noted to delaminate from the anterior follicular epithelium and move toward the oocyte. Upon arrival at the egg, they migrate a short distance dorsally toward the germinal vesicle, a PDGF-modulated process which is critical for female fertility [91].

## Clinical applications: intraovarian PRP

The breakthrough application of PRP in a reproductive context was an innovation first outlined only a few years ago [92], when a group of poor prognosis infertility patients received intraovarian injection of PRP followed by IVF with non-donor oocytes. Using autologous (conventional, non-activated) PRP in this setting was considered a logical extension of the beneficial tissue effects following standard PRP administration as documented in other clinical settings [93–95]. The rationale here was to concentrate and provide the previously described growth factors directly to a new target tissue—the adult human ovary.

Numerous cytokines, chemokines, and growth factors (e.g., hepatocyte growth factor, stromal-derived growth factor-1) have been identified as platelet products. Such platelet-derived mediators induce and modulate activation of fibroblasts and recruitment of leukocytes, neutrophils, and macrophages, resulting in elimination of dead cells and cellular debris [96]. Platelet-released factors also control proliferation and migration of other cells essential to tissue repair [72]. Angiogenesis in damaged tissue, another pivotal mechanism for tissue repair, is also regulated by platelets via release of numerous pro- and anti-angiogenic mediators upon platelet activation [97].

When autologous-activated PRP is injected into human ovarian tissue, several early observations have been noted with respect to organ function over time. Day 3 FSH/estradiol (and to a lesser extent, serum AMH) have been shown to improve following activated PRP injection into ovarian tissue [98]. While serum AMH may transiently decline (and/or FSH may briefly increase) within 2–4 weeks of intraovarian PRP injection for some patients, other recipients of ovarian autologous-activated PRP appear to skip this step and directly manifest an immediate improvement in reserve markers. The former pattern could be explained by a functional model, whereby the ovary sustains a temporary insult secondary to needle injection microtrauma associated with delivering the autologous activated PRP to the ovary. Why some patients do not demonstrate this sequence after the ovarian PRP procedure remains unknown, although this variance could be simply related to surgical/anatomical differences among PRP study patients. Additional analysis will be required to develop a more complete understanding of this recently noted ovarian tissue phenomenon.

Against this background, investigators are presented with many pieces of an unclear puzzle. The regenerative and repair processes instigated by PRP in somatic tissues remain only partially understood. PRP effects in the adult human ovary are even less known. If ovarian stem cells are indeed present, what is the risk that activated PRP might trigger tumorigenic or malignant transformations? Thus far, no adverse effects have been noted from international work using ovarian PRP, but clinical progress must be mindful of the possibility of untoward outcomes. Certainly for some poor-prognosis IVF patients, intraovarian injection of activated autologous PRP has been found to be beneficial, as frozen blastocysts and even pregnancies have been achieved without reliance on donor oocytes [98,99].

From a PRP sample preparation perspective, the role of platelet activation is likely to be important as this facilitates (and optimizes) platelet growth factor release. Commonly used techniques for platelet activation include addition of ADP [100], thrombin [101], collagen [102], Ca^++^ chloride [103], Ca^++^ gluconate [98,104], or combinations of these reagents [105–109]. Typically, platelet concentration in PRP may be up to ten times greater than ambient platelet concentration in peripheral circulation [83]. While early experience with ovarian PRP has been described [98,99], the exact mechanism of action, the role of platelet activation, and best clinical protocol have not been precisely established. For example, what platelet-derived mediators are most important in altering (improving) ovarian capacity after ovarian exposure to PRP? Do resident ovarian stem cells receive differentiation signals from PRP products as a contribution to this effect, and if so, how? Alternatively, do any oocytes harvested from PRP-treated ovaries represent a latent reserve population of dormant follicles which become eligible for recruitment following intrastromal injection of PRP? Ongoing work is designed to address each of these issues.

## Conclusion

Advanced maternal age and its associated poor ovarian reserve cannot be reliably corrected simply by gonadotropin stimulation alone. The matter of existence of adult ovarian GSCs is clearly central to activated PRP use in a reproductive setting, yet the debate is far from settled [110]. There may be sufficient evidence to include GSCs in the postnatal folliculogenesis model [111] although the traditional dogma of non-renewable, limited oocyte stores also retains serious support [112]. It could be that any putative adult ovarian GSCs are really just dedifferentiated cells which then develop as germ cells under specific *in vitro* conditions [113], as dedifferentiation has been noted in other cell types [114]. Whether or not adult ovarian GSCs represent true oogonial stem cells, and the question of whether they are clinically relevant may receive at least a partial answer from early data on IVF following intraovarian PRP dosing [98,99]. Unless some procedure can be developed to restore the native oocyte pool, continued reliance on donor oocytes for IVF will be necessary. What is has been noted from clinical work with intraovarian PRP may be regarded as an echo of ancestral growth factor functions, iterated in related sets of morphogenetic processes during evolutionary development [115]. Our understanding of the activated PRP substrate, its derivative growth factors, putative receptor targets, differentiation regulators, as well as other aspects of this innovative approach are at a nascent stage [116]. It is plausible that any improved ovarian function observed after exposure of adult ovarian tissue to PRP components is merely a manifestation of precursor cell differentiation [117,118], evoked by still poorly understood growth signals of platelet origin.

## Abbreviations

ALDH1: aldehyde dehydrogenase 1
BMP: bone morphogenetic protein
CD133: cluster of differentiation 133
DPPA3: developmental pluripotency associated 3
GFP: green fluorescent protein
GSC: germline stem cell
IFITM3: interferon-induced transmembrane protein 3
LY6A: lymphocyte antigen 6 complex, locus A
PDGF: platelet-derived growth factor
PGC: primordial germ cell
PRP: platelet-rich plasma
SMC: smooth muscle cell
Sox2: SRY box 2
SSEA: stage-specific embryonic antigen
Stra8: stimulated by retinoic acid 8
TCF: T-cell factor
TGF-β: transforming growth factor β
Utf1: undifferentiated embryonic cell transcript factor-1
VSEL: very small embryonic-like stem cell
Zfp296: zinc finger protein 296
